# An Unexpected Complication Resulting from Radiofrequency Ablation for Treating Facet Joint Syndrome: A Case Report

**DOI:** 10.3390/medicina59111996

**Published:** 2023-11-14

**Authors:** Hyung-Sun Won, Shin-Hyo Lee, Young Jean Ahn, Miyoung Yang, Yeon-Dong Kim

**Affiliations:** 1 Department of Anatomy, Wonkwang University School of Medicine, Iksan 54538, Republic of Korea; hswon01@wku.ac.kr (H.-S.W.); shinhyolee1@wku.ac.kr (S.-H.L.); 2 Jesaeng-Euise Clinical Anatomy Center, Wonkwang University School of Medicine, Iksan 54538, Republic of Korea; 3 Department of Anesthesiology and Pain Medicine, Wonkwang University Hospital, Wonkwang University School of Medicine, Iksan 54538, Republic of Korea; yjahn95@naver.com; 4 Sarcopenia Total Solution Center, Wonkwang University School of Medicine, Iksan 54538, Republic of Korea; 5 Wonkwang Institute of Science, Wonkwang University School of Medicine, Iksan 54538, Republic of Korea

**Keywords:** burn, chronic pain, complication, facet joint, lumbosacral region, needle, nerve block, radiofrequency ablation, spinal pain, surgery

## Abstract

Lumbar facet joints have been identified as a potential source of chronic low back pain (LBP) in 15% to 45% of patients, with the prevalence of such pain varying based on specific populations and settings examined. Lumbar facet joint interventions are useful in the diagnosis as well as the therapeutic management of chronic LBP. Radiofrequency ablation (RFA) of medial branch nerves is recognized as a safe and effective therapy for chronic facet joint pain in the lumbosacral spine, and its efficacy has already been established. The use of RFA is currently widespread in the management of spinal pain, but it is noteworthy that there have been works in the literature reporting complications, albeit at a very low frequency. We present a case of third-degree skin burns following radiofrequency ablation (RFA) for the management of facet joint syndrome. Postoperatively, the patient’s skin encircling the needle displayed a pallor and exhibited deterioration in conjunction with the anatomical anomaly. The affected area required approximately 5 months to heal completely. During RFA, heat can induce burns not only at the point of contact with the RF electrode but also along the length of the needle. Vigilant attention is necessary to ensure patient safety and to address any potential complications that may arise during the procedure, including the possibility of minor technical errors.

## 1. Introduction

Spinal pain is a multifaceted phenomenon characterized by challenges in its precise definition arising from diverse structures within the spinal framework. These complexities contribute to the uncertainty surrounding clinical evidence pertinent to spinal pain management. Predominantly, lower back pain (LBP) emerges as the paramount manifestation of spinal discomfort, distinguished by its chronic nature and varying degrees of severity. Diagnostic and therapeutic interventional techniques have been established in terms of validity and effectiveness, emphasizing the significance of structural interventional pain management [1]. Among the intricate components of spinal architecture, facet joints are duly acknowledged as one of the primary contributors to the genesis of LBP. Therefore, interventions targeting facet joints are frequently used in clinical practice. Representatives among these are lumbar facet joint steroid injections, lumbar medial branch blocks, and radiofrequency ablation (RFA), targeting the medial branches of lumbar spinal nerves.

As early as the 1950s, RFA has been employed as a clinical approach for managing chronic pain [2]. It is distinctive in its ability to achieve dependable and precise thermal lesioning in a specific neurological site of interest by utilizing the energy of a low-energy, high-frequency alternating current, thereby generating an electrical field between the active electrode and the grounding pad. The end result can produce pain relief for a prolonged duration of up to 6 months or longer [3]. Numerous randomized controlled trials and observational studies have already underscored the efficacy of RFA in treating spinal pain, advocating it as a safer and more judicious alternative when compared to other treatment modalities [4]. However, it is imperative to initiate the RFA procedure with a meticulous assessment of the patient’s pain response.

The execution of the RFA procedure necessitates adeptness and experience in the manipulation and oversight of the equipment to minimize potential complications. Technically, the inadequate placement of an electrode or grounding pad could engender a reduction in therapeutic efficacy due to an incomplete circuit formed amidst body tissues. Complications following RFA are uncommon, with only a few documented cases pertaining to needle-related issues reported in the literature.

Consequently, we present a unique case of third-degree skin burns localized at the site of percutaneous RF electrode application, stemming from RFA conducted to address the lumbar facet joint syndrome. This unexpected manifestation required extensive wound management spanning several months following the procedure.

## 2. Case Presentation

**Figure 1:**
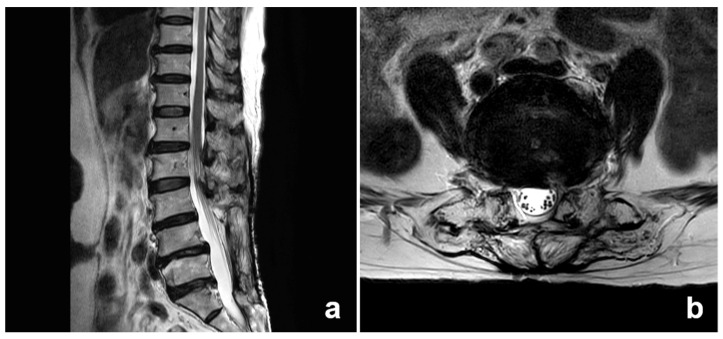
Magnetic resonance (MR) images obtained from the patient. (**a**) Sagittal and (**b**) Axial images show that the metal fixations at the L4/5 level are completely removed by the previous surgery and there are also not any neurological abnormalities.

A 69-year-old woman (162 cm in height, 52 kg in weight) sought medical attention at our hospital, citing LBP that had worsened over a span of 3 years. The patient complained of discomfort during lumbar flexion and extension, with the pain localized to the left side of the dorsal region, although episodes of radiating pain occasionally extended to the ipsilateral posterior thigh. Importantly, no neurological impairments were apparent. Her subjective assessment, quantified using the visual analog scale (VAS), yielded a pain intensity of 7 out of 10. Notably, the patient underwent posterior lumbar interbody fusion at the L3–5 vertebrae 6 years prior, a historical intervention for lumbar spondylolisthesis. Postoperative outcomes proved unsatisfactory, prompting a second surgery for metallic implant removal 3 years prior to the current presentation. In spite of the previous two surgical procedures, she continued to experience enduring, severe localized pain in the lower left back, situated to the side of the midline and radiating outward without any accompanying radicular symptoms. Imaging studies, including magnetic resonance imaging (MRI), revealed no neurological abnormalities (Figure 1), and there were no other conditions requiring surgical interventions.

Aside from hypertension and osteoporosis, the patient had no other significant medical conditions. Despite various conservative treatments, including medication therapy, she continued to complain of persistent pain. After confirming the MR images, a diagnostic medial branch block (MBB) was decided to determine whether the patient’s symptoms were caused by the facet joint. The MBB was applied to the left L4/5 and L5/S1 levels, respectively. It was performed with a 25 g needle, 90 mm long, and 5 mg of dexamethasone and 0.5 mL of 0.5% mepivacaine were injected. The diagnosis of lumbar facet joint syndrome was ascertained after the confirmation of a >50% pain reduction in VAS following two diagnostic lumbar medial branch blocks conducted at 2-week intervals. The entirety of both procedures were performed under fluoroscopy, and no adverse events were observed.

**Figure 2:**
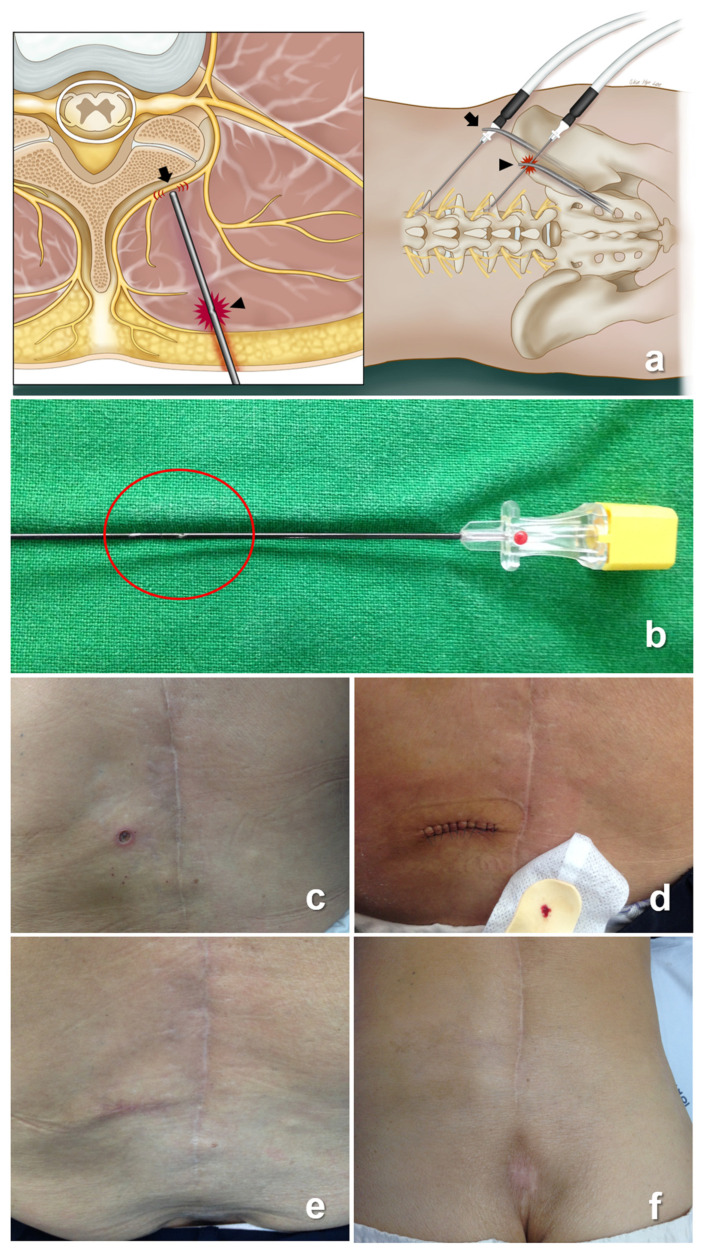
Images display each condition for the present case. (**a**) Performing RFA using hemostatic forceps with needle hub (arrows) and shaft (arrowheads), the former has an intended effect, while the latter can occur as an unexpected side effect due to some damage to the needle shaft. (**b**) Finding incidentally that some part of a needle is uninsulated after finishing the procedure; (**c**) Finding third-degree skin burns in the patient; (**d**) Applying surgical care to the patient by a plastic surgeon; (**e**) Observing the wound repair process; (**f**) Wound recovered completely.

To optimize pain management outcomes, our strategy encompassed the implementation of RFA subsequent to obtaining patient consent. After applying 1% lidocaine for local anesthesia, the RF needle (22-G, 10 cm in length, curved, with a 10 mm active tip) was introduced into the designated target site under fluoroscopic guidance. The needle tip underwent iterative adjustments to ascertain the ideal position. Owing to suspected tissue scarring from previous surgeries, manual needle manipulation was challenging, prompting the use of hemostatic forceps during certain phases of the procedure (Figure 2a). As the RF needle neared the target position, the stylet was extracted, and the RF probe was subsequently introduced. The definitive placement of the RF probe was established through sensory stimulation (50 Hz) or the patient experiencing a tingling sensation at a voltage below 0.5 V. If the threshold value exceeded 0.5 V, the needle was carefully advanced until the patient felt sensory stimulation. After confirming the optimal needle position through these processes, the preliminary numbing of the tissues was achieved via the injection of 0.3 mL of 2% lidocaine in preparation for thermal ablation. The target medial branch was then subjected to a 90 s lesioning process at a temperature of 75 °C. However, inadvertently, we found that some portions of the needle were uninsulated after the procedure (Figure 2b).

The patient reported no discomfort during the RFA procedure. However, they experienced moderate lumbar pain postoperatively despite the absence of a discernible lesion. Following the procedure, on the second day, the patient intended to visit our hospital earlier than scheduled because of persistent lumbar pain. However, due to personal circumstances, her visit was delayed by 7 days. Upon examination, a skin lesion measuring 20 mm in diameter was identified and ultimately considered a third-degree burn (Figure 2c). The swift intervention was ensured and orchestrated by a plastic surgeon, including dressing, debridement, and the suturing of the affected skin (Figure 2d). After the removal of the suture thread, the wound exhibited complete recovery without any aberrations (Figure 2e,f). The comprehensive timeline encompassing the management of this unexpected skin burn extended over a duration of 5 months. However, the preexisting LBP was effectively controlled, as evidenced by a VAS score of 2 or 3 points, with an analgesic applied after the RFA procedure. Importantly, this level of pain management was unrelated to any skin complications.

The publication of the present case was approved by the institutional review board of Wonkwang University Hospital (IRB ID No. WKUH 2023-08-013), and written informed consent was obtained from the patient.

## 3. Discussion

RFA refers to the application of a high-frequency electrical current, administered through an RF electrode, to the patient’s tissue, eliciting a biological response that includes the thermal incapacitation of nerves responsible for transmitting nociceptive signals [5]. To explain the process in more detail, an insulated needle first conducts a high-frequency electrical current. And this current generates an electric field at the needle’s tip, inducing molecular movement that results in the production of thermal energy. The heat emanating from the tip of the RFA device is precisely directed to form a minor lesion within a nerve, thereby interrupting the transmission of pain signals. Well-designed sham-controlled randomized clinical trials concerning lumbar facet joints offer strong evidence supporting the effectiveness of continuous conventional RF ablation in alleviating lumbar facet joint pain [6]. Nonetheless, the clinical significance of the achieved pain relief and its duration remains uncertain, likely due to variations in the denervation techniques employed. Presently, the optimal technique is deemed to entail the positioning of the needle as parallel to the medial branch, which is feasible [7,8]. Consequently, RFA has found frequent application in the denervation of medial branches of the lumbar spine, which are widely employed to treat facet-mediated LBP [9]. The majority of RF-related complications usually have a minimal impact and are typically transient, primarily involving postprocedural pain and neuritis. Deafferentation pain and the formation of a neuroma were reported to be caused by chemical, surgical, and cryoablation neurolysis and, more recently, post-RFA as well [10].

The atrophy of the multifidus muscle is known to be a complication of denervation. On the other hand, camptocormia has recently been identified as an immediate complication of RFA [11]. However, the comprehensive documentation of RFA-associated complications is rare. It remains uncertain whether this scarcity is a product of the infrequent occurrence of such complications or a failure to report these types of complications. Predominant among the complications affiliated with RF are those related to needle placement and complications arising from neurolysis. The majority of these difficulties tend to be transient and short-lived, encompassing localized swelling and pain at the site of needle insertion. Generalized complications may include pneumothorax, dural puncture, spinal cord trauma, a subdural injection, neural trauma, and hematoma formation, whereas infectious complications can entail abscess formation and bacterial meningitis [12]. Minor complications encompass various symptoms, such as flushing, hypotension, non-postural headaches, exacerbated pain, reduced sensation, and allodynia in the paravertebral skin or denervated facets [12]. Based on the literature review, the prevalence of minor complications, such as pain or temporary neuritis after RFA, is documented to be less than 1% [13]. It is pertinent to note that these complications are reported to have little correlation with the damage caused by the needle itself. When looking at the aspect of a mechanical configuration in the RF generator, monopolar RF systems employ grounding pads to establish the completion of the RF circuit. Incidents of thermal injuries at the site of the grounding pad have been increased by the application of higher currents, such as the cooled RF treatment for malignant tumors [14].

Regarding the RFA procedure, the attainment of an optimal diagnostic and therapeutic outcome is inextricably tied to the precise positioning of the needle under fluoroscopic guidance. However, during this procedure, judicious attention must be paid to radiation exposure, which affects both the patient and the physician. Typically, needle manipulation is performed manually. Nonetheless, cases akin to ours, where structural modifications stemming from prior surgical interventions introduce complications, may necessitate the use of specialized instruments (i.e., hemostatic forceps). Under these exceptional circumstances, mechanical forces exerted during the procedure may compromise the integrity of the needle, leading to scenarios where the needle becomes bent or uninsulated. In particular, an uninsulated needle can cause electrical burns when encountering soft tissues, including the skin, subcutaneous tissue, and musculature. This is attributable to the fact that thermal stimulation is not only limited to the needle tip but also extends to encompass the uninsulated regions. In such circumstances, it is essential to consider that even a minor, unexpected injury can cascade into serious complications unrelated to spinal pain. In the cases of cardiac RFA and solid tumor RFA procedures reported previously, an unexpected side effect such as skin burns could be caused by the grounding pad, and its prevalence rate was mentioned from 5% to 33% for first-degree burns and from 0.1% to 3.2% for second- or third-degree burn [14]. The inadequate positioning of a grounding pad, defective pad design, inadequate contact between the pads and the patient, disruptions in the electrical circuit, and profound levels of anesthesia could be associated with these kinds of injuries [15]. However, to our knowledge, there was no report of a third-degree burn by a needle-related injury during RFA treatment for facet-mediated spine pain. The present case highlights the importance of a meticulous patient selection process for RFA procedures. If postoperative adhesions or structural changes exist at the site of needle insertion, special attention should be given during manipulating an electrode or needle, and it may be challenging for safer procedures. In the event of a burn, the following steps for management are highly desirable for the patient’s condition: (1) implementing a thorough and long-term follow-up; (2) providing patient education and support; and (3) administrating appropriate wound care. Consulting with a plastic surgeon or dermatologist can also be considered as a part of managing these complications. Consequently, a careful procedural approach is required, including meticulous pain assessment and continuous monitoring of the RF machine components.

In addition, the present case report has a noteworthy limitation. High-quality controlled trials on lumbar facet joints offer strong evidence for the efficacy of RFA in reducing pain. However, the clinical relevance and duration of pain relief remain unclear, likely due to variations in denervation techniques [16,17]. It is also essential to note the lack of standardized continuous RFA protocols for specific targets, which complicates the comparison of RF complication rates across various studies addressing the same pain syndrome.

## 4. Conclusions

RFA enables the precise lesioning of nerves and neurological structures responsible for transmitting pain signals to the central nervous system. It has demonstrated itself as a crucial resource in the management of chronic pain. Most of the common complications associated with RFA procedures appear to have low incidence and minimal consequences and are usually temporary. It is uncertain whether this is linked to the low incidence of complications associated with RFA procedures or a lack of reporting of such complications by pain providers. It should also be noted that there is a possibility of undisclosed complications occurring, as in this case report. Appropriate patient selection and precautions guided by the interventional physician are in place, and the advantages of RFA procedures appear to significantly outweigh the risks.

## Data Availability

All the data are available from the corresponding author upon reasonable request.
